# Mental health literacy among pediatric hospital staff in the United Arab Emirates

**DOI:** 10.1186/s12888-017-1556-z

**Published:** 2017-12-08

**Authors:** Nabeel Al-Yateem, Rachel Rossiter, Walter Robb, Alaa Ahmad, Mahmoud Saleh Elhalik, Sumaya Albloshi, Shameran Slewa-Younan

**Affiliations:** 1 Department of Nursing, College of Health Sciences, University of Sharjah, P O Box 27272, Sharjah, United Arab Emirates; 2 School of Nursing, Midwifery and Indigenous Health, Faculty of Science, Charles Sturt University, Orange, Australia; 3 Research Institute for Medical and Health Sciences (RIMHS), University of Sharjah, Sharjah, United Arab Emirates; 4 School of Nursing, Midwifery & Indigenous Health Charles Sturt University, Orange, Australia; 5 Menzies Health Institute, Griffith University, Brisbane, Australia; 6 Shaikh KHalifa Medical City (SKMC), Abu Dhabi, United Arab Emirates; 7 Department & Neonatology Unit, Latifa Hospital, Dubai, United Arab Emirates; 8 Ministry of Health and Prevention, Dubai, United Arab Emirates; 9 Mental Health, Centre for Health Research, School of Medicine, Western Sydney University, Sydney, Australia; 10 Centre for Mental Health, Melbourne School of Population and Global Health, University of Melbourne, Melbourne, Australia

**Keywords:** Cross-cultural care, Early intervention, Health literacy, Health promotion, Mental health, Professional practice gaps, Pediatric care

## Abstract

**Background:**

In the United Arab Emirates (UAE) 35% of the population are aged 0–24 years. A significant proportion of these young people are living with chronic conditions (e.g., asthma, type 1 diabetes, cardiac conditions, and genetically-transmitted conditions such as thalassemia and cystic fibrosis). This group has increased vulnerability to developmental delays and mental health problems, and is increasingly coming to the attention of service providers in mainstream schools, primary healthcare centers, and pediatric hospitals. Despite the government directing attention to improving the mental health of the UAE population, there is concern that mental health services are not growing at the rate needed to meet the mental health needs of children and young people with chronic conditions.

**Method:**

A cross sectional survey design was used to determine the mental health literacy of nurses and other healthcare professionals working with children with chronic illnesses. Participants completed a culturally-adapted mental health literacy questionnaire comprising three vignettes of fictional characters meeting diagnostic criteria for posttraumatic stress disorder, psychosis, and depression with suicidal thoughts. Participants also completed the Kessler Psychological Distress Scale (K10).

**Results:**

Participants were 317 healthcare professionals from across the UAE. The majority were nurses. Correct identification of the diagnosis for each vignette was limited, with the highest level of accuracy achieved for the psychosis vignette (*n* = 113, 54.3%). Accurate identification of appropriate evidence-based interventions was also limited. K10 scores indicated 40% of participants had moderate to high levels of psychological distress.

**Conclusions:**

These findings are concerning and provide important data to inform the development of undergraduate and continuing education programs for nurses. The K10 scores suggest healthcare professionals are under considerable stress, highlighting the need to support healthcare professionals who experience multiple psychosocial stressors.

## Background

The United Arab Emirates (UAE) is an Arabian Peninsula nation formed in 1971 from seven sheikdoms. It is currently ranked as one of the top 25 countries worldwide in population growth [1]. Despite rapid population growth and the relative wealth of the nation, there are concerns that mental health services are not growing at a rate that matches the country’s needs [2]. In 2010, the UAE government prioritized mental health as one of the top five health priority areas requiring attention [3]. Another pressing concern directly linked to the UAE’s rapid population growth is the need to optimize care for young people aged 0–24 years, who comprise approximately 35% of the total population [1]. Improvements in the quality of healthcare and a marked decrease in infant mortality have resulted in a growing number of children with chronic conditions (e.g., asthma, type 1 diabetes, cardiac conditions, and genetically-transmitted conditions such as thalassemia and cystic fibrosis) who are able to actively participate in the community. However, children living with chronic conditions face multiple challenges that may impede or delay their achievement of developmental and social milestones [4]. Challenges include repeated hospitalizations, ongoing periods of poor health, decreased physical strength and skills, or changes in appearance due to illness [4, 5]. Improved health outcomes and the multiple challenges experienced by children living with chronic illness mean that community service providers (e.g., mainstream schools, primary healthcare centers, and hospital-based pediatric services) need to provide enhanced support to enable these children to achieve their full potential.

Children and adolescents living with chronic physical health conditions have increased vulnerability to mental health problems such as anxiety and depression [6–12] [6–12] [6–12] [6–12] Undetected mental illness in children or adolescents further disrupts the achievement of developmental tasks required for optimal health and vitality in adulthood [6–12]. Early detection of mental illness and referral to appropriate treatment is essential to avoid more serious mental illness in future. Therefore, health professionals caring for this age group need adequate knowledge and understanding of the nature and treatment of mental health problems; that is, they must demonstrate sufficient *mental health literacy* (MHL) to identify emerging mental illness, provide information to the child/young person and their carers, and make referrals to appropriate services. Although mental health services have been identified as requiring further development, Sayed [13] reported that well-established, comprehensive in- and outpatient mental health services are co-located with hospitals in major UAE cities, and other mental health services are accessible via educational and community settings. However, community-based mental health services staffed with specialist mental health clinicians are limited.

MHL is defined as “knowledge and beliefs about mental disorders which aid their recognition, management or prevention” [14]. MHL encompasses: a) the ability to recognize specific disorders; b) knowledge of how to seek mental health information; c) knowledge of risk factors and causes, self-help treatments, and professional help; and d) attitudes that promote recognition and appropriate help-seeking. Research has demonstrated that improved levels of MHL can achieve two key outcomes. First, MHL empowers the recipient community with an understanding of mental disorders that facilitate prevention, early intervention, and treatment. Second, individuals with a sound level of MHL have the means by which they are able to make informed decisions about accessing mental healthcare [15].

To our knowledge, no study has examined MHL among pediatric health professionals working in hospitals in the UAE. Measuring MHL in this cohort may inform development of focused mental health training programs for health professionals in the UAE, and more broadly inform mental health promotion programs. This study aligns with the priority on the ongoing development of evidence-based mental health services in the UAE, based on research evidence conducted within the country. Given the paucity of literature, no “a priori” hypotheses were determined. However, this study aimed to determine knowledge of, and beliefs about, helpfulness of treatment interventions and providers of care for three common mental health conditions (posttraumatic stress disorder [PTSD], depression with suicidal thoughts, and psychosis) among healthcare professionals working in pediatric hospital settings in the UAE. A large proportion of the healthcare workforce in the UAE is expatriate; many are separated from their families or have come from countries affected by sociopolitical upheaval. Therefore, a secondary objective of this study was to explore potential associations between healthcare professionals’ level of distress and their ability to identify these three mental health conditions. A cross-sectional measure of non-specific psychological distress in the target population was administered to allow this exploration.

## Methods

### Study design and participant recruitment

A cross sectional study design was used. Participants were recruited from all emirates in the UAE. Initially, a clustered sampling strategy was proposed to obtain a representative sample from the seven UAE emirates and each health sector. All government (*n* = 33) and private (*n* = 71) hospitals were identified [16]. From these 104 hospitals, the main government and private providers of pediatric care in each emirate (*n* = 13) were identified and contacted to regarding access to potential participants. However, accessing potential participants presented significant challenges and resulted in a change to the planned sampling strategy.

The fragmented nature of UAE healthcare services required ethics approval from multiple parties to enable access to potential participants. Therefore, access to potential participants employed in governmental hospitals involved lengthy administrative procedures. In addition, access to all private hospitals was refused. Given these challenges, the sampling procedure used was sampling of the total accessible population. Thirteen potential hospitals were identified, and approval to conduct this study was obtained from seven hospitals. The participating hospitals represented six emirates: Sharjah (*n* = 1); Dubai (*n* = 2); Abu Dhabi (*n* = 1); Ajman (*n* = 1); Ras Al-Khaimah (*n* = 1); and Umm Al-Quwain (*n* = 1). These sites were government hospitals or hospitals managed by independent authorities, such as the Health Authority of Abu Dhabi, and the Dubai Health Authority. The non-participating emirate represents a small proportion of the total UAE population.

All nurses and doctors who provided care for children and adolescents with chronic illnesses in different clinical settings (out- and inpatient departments) in the seven participating hospitals were invited to participate in the study. Data were collected via paper-based or online questionnaires, according to the preference of the participating hospitals. Data collection was coordinated centrally through a liaison person in each of the participating clinical settings. The questionnaire (or link to the online questionnaire) was delivered to these liaison persons, who distributed questionnaires to participants and collected completed questionnaires. Because of this centralized administration process, the specific number of staff in each setting and the number of distributed versus returned questionnaires could not be accurately determined. However, it is estimated that a maximum of 1400 health professionals (e.g., nurses and doctors) were employed in the participating pediatric settings (i.e., those that granted access). In total, 379 questionnaires were returned; assuming that 1400 questionnaires (hard copy or online link) were made available to clinicians, the estimated response rate was 27%. Response rates were not consistent across the sites or professions. However, the largest hospitals returned the majority of the questionnaires, and the response rate from nursing staff was proportionally significantly higher than that from medical staff. Despite these challenges, the final sample represents the broad spectrum of the socioeconomic demographic of the overall population and healthcare systems across the UAE, with the exception of private hospitals.

### Measures

#### MHL survey

This study used an adapted version of the MHL questionnaire as reported by Jorm and colleagues [14] and later modified by Slewa-Younan [17], after obtaining permission from those authors. Further modifications to the MHL questionnaire for this study were undertaken by two authors (NA, RR). Specifically, the vignettes were adapted based on the consensus of authors (NA, RR) experienced in working in pediatric health settings in the UAE. Care was taken to ensure the vignettes were culturally valid and reflected appropriate diagnostic criteria for the three disorders, according to the Diagnostic and Statistical Manual of Mental Disorders (DSM) Fifth Edition [18]. Modifications included changing the names in the vignettes to Arabic names (i.e., Miriam, Abdul, and Saed), and amending some phrases (e.g., “Reading Bible” was changed to “Reading Koran or Bible” and “Talking with priest” to “Talking with religious person or priest”). English is the language common to all healthcare professionals in the UAE health system. Although Arabic is the official language in the UAE, translating the questionnaire into Arabic would have excluded a significant number of expatriate healthcare professionals from different linguistic backgrounds, and required cross-translation expenses outside the available funding.

The questionnaire was piloted with 12 final-year Bachelor of Health Science (nursing) students to determine cultural acceptability. These students were representative of the cross-cultural backgrounds and spoken languages of the target population for this study (i.e., Arabic, Indian, and Filipino). No changes were required following the pilot testing.

The three case vignettes presented were fictional characters. The first (Miriam) suffered from PTSD, the second (Abdul) experienced depression with suicidal thoughts, and the third (Saed) displayed symptoms indicative of psychosis. Each vignette was followed by a series of questions addressing the nature and treatment of the problem described, including problem recognition and beliefs about the likely helpfulness of various treatment options and treatment providers (see the Appendix for the vignettes).

#### General psychological distress

The Kessler Psychological Distress Scale (K10) [19] was used to assess participants’ non-specific psychological distress. The K10 is a self-report questionnaire designed to assess psychological distress in relation to anxiety- and depression-related symptoms. The K10 has good psychometric properties, with an internal consistency (Cronbach’s alpha) of 0.86 reported for Arabic speaking populations [20]. The Cronbach’s alpha in this study was 0.95. K10 scores range from 10 to 50, with higher scores indicating greater distress. This study used the established thresholds of low to mild (10–21), moderate (22–29), and severe distress (≥30) to classify the level of psychological distress reported by participants.

Providing follow-up or support for participants with K10 scores indicating psychological distress was not possible as all data were anonymized. However, information about the study provided with the questionnaire included information about referral to psychological services.

### Statistical analyses

Statistical analyses were used to test the effect of sociodemographic characteristics and K10 levels on problem recognition and beliefs about interventions. These tests were based on the research hypotheses and were determined before considering the data. Statistical analysis was performed using the freely available R software version 3.2.2 [19].

The chi-square test of independence was used for categorical sociodemographic variables (sex, years of residence in the UAE, language group, and region of origin) (*p* < 0.05), based on the hypothesis that responses for problem recognition and beliefs about interventions were independent of sociodemographic variables. The Kruskal-Wallis test (p < 0.05) was used for numerical sociodemographic variables (age and years of experience) and K10 score, to test the hypothesis that the variable was not significantly associated with problem recognition and beliefs about interventions. This was because a normal distribution could not be assumed, particularly when numbers of responses for problem recognition and beliefs about interventions categories were small. Pairwise post hoc comparisons of significant sociodemographic characteristics were performed using Dunn’s procedure with a Bonferroni correction for multiple comparisons, to determine which response categories had significantly different values.

#### Missing values

Missing values for demographic variables are shown in Table 1. In calculating K10 scores, if one or two of the 10 items were missing, they were estimated as the average of the available data points. Records with more than two missing values received an overall K10 score of “missing.” Cases with missing values were automatically excluded from the analyses.

**Table 1:** Demographic characteristics of the study participants collapsed according the MHL surveys completed

		PTSD		Depression with suicidal thoughts		Psychosis	
		N	%	n	%	n	%
All participants		317	100	281	100	208	100
Gender	Female	274	86.4	243	86.5	181	87.0
	Male	33	10.4	33	11.7	25	12.0
	Missing	10	3.2	5	1.8	2	1.0
Age group	20–29	79	24.9	64	22.8	45	21.6
	30–39	129	40.7	122	43.4	98	47.1
	40–49	74	23.3	69	24.6	47	22.6
	50–59	18	5.7	17	6.0	13	6.3
	60+	2	0.6	2	0.7	2	1.0
	Missing	15	4.7	7	2.5	3	1.4
Region	Middle east	40	12.6	37	13.2	25	12.0
	Africa	13	4.1	13	4.6	9	4.3
	Sub-continent	119	37.5	117	41.6	98	47.1
	South East Asia	23	7.3	22	7.8	19	9.1
	UAE	32	10.1	23	8.2	12	5.8
	Missing	90	28.4	69	24.6	45	21.6
Years of residency	9 or less	81	25.6	80	28.5	71	34.1
	10–19	53	16.7	51	18.1	39	18.8
	20–29	40	12.6	35	12.5	23	11.1
	30+	40	12.6	38	13.5	30	14.4
	Missing	103	32.5	77	27.4	45	21.6
Language	Arabic	84	26.5	72	25.6	47	22.6
	English	35	11.0	34	12.1	29	13.9
	India	84	26.5	81	28.8	67	32.2
	Philippines	20	6.3	19	6.8	16	7.7
	Other	6	1.9	7	2.5	0	0.0
	Missing	88	27.8	68	24.2	49	23.6
Profession	Medicine	27	8.5	14	5.0	6	2.9
	Nursing	290	91.5	267	95.0	202	97.1
Qualification	Nursing Diploma	116	36.6	114	40.6	85	40.9
	Nursing BSc	75	23.7	73	26.0	65	31.3
	Nursing post-graduate qualification	11	3.5	11	4	8	3.9
	Medical BSc	13	4.1	3	1.1	2	1.0
	Medical Post-graduate qualification	9	2.9	8	2.8	2	1.0
	Other	1	0.3	1	0.4	0	0.0
	Missing	92	29.0	71	25.3	46	22.1
Years of experience	0- < 5	53	16.7	47	16.7	37	17.8
	5- < 10	81	25.6	70	24.9	55	26.4
	10- < 15	48	15.1	45	16.0	35	16.8
	15- < 20	51	16.1	46	16.4	31	14.9
	20+	68	21.5	65	23.1	46	22.1
	Missing	16	5.0	8	2.8	4	1.9
Work Area	Emergency Department	17	5.4	17	6.0	14	6.7
	Out Patient Department	164	51.7	152	54.1	125	60.1
	Pediatric wards	112	35.3	91	32.4	52	25.0
	Pediatric Intensive care unit	10	3.2	11	3.9	9	4.3
	Missing	14	4.4	10	3.6	8	3.8
K10 scores	Mean	21.8					
	SD	9.5					
K10 ranges	10- < 22	209 (59.9%)					
	22- < 30	78 (22.3%)					
	30–50	62 (17.8%)					

### Ethical considerations

Ethics approval was obtained from the University of Sharjah Research Ethics Committee (Ref#: ERC/23/11/15/46), Dubai Scientific Research Ethics Committee (Ref#:DSREC-12/2015_13), and Ministry of Health Research Ethics Committee (Ref#:R04). Return of the questionnaire was considered as provision of consent to participate in the study.

## Results

### Sample characteristics

In total, 379 healthcare professionals responded to the survey. Of these, 370 had useable responses to K10 items, 324 had responded to at least one of the clinical MHL vignettes, and 315 had responded to at least one vignette plus the K10 questions. The majority of participants were nurses (92.9%); the remainder was medical doctors (7.1%). Most participants were females (90.7%) and aged 30–39 years (43.4%). A large proportion of participants were from the Indian sub-continent; followed by the Middle East, UAE nationals, South East Asia, and Africa; over one-third (38.3%) did not report their nationality. The main spoken languages were Arabic (37.2%) or an Indian language (35.9%), and 15.4% reported English as their mother tongue. A majority (58.5%) of nurse participants had a diploma-level qualification and 59.1% of participating medical doctors had a bachelor’s degree. Finally, 83.1% of all participants had at least 5 years of experience, and 53.7% worked in outpatient departments (OPDs). The mean K10 score was 21.9 (SD 9.5); 59.9% of participants (*n* = 209) had a K10 score in the mild range (10–21), 22.3% (*n* = 78) in the mid-range (22–29), and 17.8% (*n* = 62) in the high range (≥30).

Participants’ demographic characteristics according to completed MHL vignettes are presented in Table 1.

### PTSD: Miriam

Participants were asked “What would you say is Miriam’s main problem?” In total, 149 (47.0%) chose PTSD, 65 (20.5%) chose depression, and 65 (20.5%) thought the character was suffering from fear. Collectively, this accounted for 88.0% of all responses.

Table 2 shows the percentage of respondents who considered interventions in each subcategory (treatment activities, medicines, or people) as “helpful,” “harmful,” or “neither” for the problem described. “Getting information about the problem” was the treatment activity most commonly considered helpful (87.0%), followed by “Reading the Koran or Bible” (84.4%) and “Getting out and about” (71.9%). “Psychotherapy focused on changing thoughts and behavior” was selected as the most helpful treatment activity by 37.4% of participants. “Anti-depressant medication” was the medication most commonly noted as helpful (56.9%), and was considered the most helpful medication by 44.3% of participants. In terms of assistance from people, participants frequently considered psychologists (83.0%), psychiatrists (77.5%), and family members (75.5%) to be helpful. Psychologists and psychiatrists were each regarded as most helpful by 29.9% of participants.

**Table 2:** Perceived helpfulness of interventions for PTSD vignette (*n* = 317)

Interventions	Helpful	Harmful	Neither	Most helpful^a^
Treatments and activities				
Getting information about the problem and available services	87.0	10.5	2.5	13.9
Reading the Koran or Bible	84.4	11.7	3.8	9.5
Getting out and about more/finding some new hobbies	71.9	25.5	2.6	3.4
Psychotherapy focusing on changing thoughts and behaviours (cognitive behaviour therapy)	70.1	27.7	2.2	37.4
Have a prayer session or reading with a religious leader	68.6	30.4	1.0	3.1
Just talking about the problem (e.g. to a family member or close friend)	66.3	30.2	3.5	5.4
Relaxation (e.g. having a massage)	66.0	33.3	0.6	3.4
Psychotherapy focusing on relationships with others	64.2	33.9	1.9	1.7
Psychotherapy focusing on causes that stem from the past	61.4	34.4	4.2	13.9
Reading a self-help book	53.0	42.1	4.9	0.0
Improving diet and/or getting more exercise	52.1	46.7	1.3	1.4
Hypnosis	19.9	69.0	11.1	0.7
Trying to deal with the problem on her own	18.8	47.1	34.1	2.0
Admission to a psychiatric hospital	18.6	61.1	20.3	3.7
Traditional therapies (e.g. herbs, honey, black cumin seed, olive oil, dates, cupping - Hijama)	16.7	80.1	3.2	0.3
Drinking alcohol to relax	2.5	23.1	74.4	0.0
Medicine Type				
Anti-depressant medication (e.g. Prozac)	56.9	8.2	34.9	44.3
Medication to help you relax (e.g. Xanax, Valium)	51.9	8.4	39.6	43.0
Vitamins and minerals (e.g. Vitamin C)	45.1	0.0	54.9	12.7
Person/Service				
Psychologist	83.0	2.0	15.1	29.9
Psychiatrist	77.5	1.0	21.6	29.9
Family member	75.5	2.3	22.3	16.5
Religious person or priest	74.2	1.9	23.9	5.2
Community mental health worker/team (e.g. social worker, mental health nurse)	71.8	1.0	27.2	4.1
Close female friend	65.0	1.9	33.1	7.2
Homeland social group/club	62.8	5.5	31.7	3.1
Family or local doctor	58.5	2.0	39.5	2.1
Community religious organization	50.2	2.0	47.8	0.0
Telephone counseling	38.4	4.2	57.3	1.0
Close male friend	16.6	8.6	74.8	1.0

^a^Percentage of sample rating the specific intervention item as ‘the most helpful’ for treating problem described in vignette

### Factors affecting responses to vignette questions

The only factor associated with a correct diagnosis of PTSD was participants’ work area. Those who worked in acute areas (e.g., emergency departments and intensive care units) were significantly better at correctly selecting the diagnosis of PTSD than those in OPDs and pediatric wards (*P* = 0.033).

### Depression with suicidal thoughts: Abdul

Participants were asked “What would you say is Abdul’s main problem?” In total, 151 participants (53.7%) chose depression with suicidal thoughts, and 87 (31.0%) chose depression, which accounted for 84.7% of all responses. Eleven participants (3.9%) thought Abdul was suffering from fear, and 11 (3.9%) thought he was suffering from “no real problem, just a phase.”

Table 3 shows the percentage of participants who considered interventions in each subcategory (treatment activities, medicines, or people) as “helpful,” “harmful,” or “neither”’ for Abdul’s problem. “Getting more information” was the treatment activity most commonly considered helpful (90.6%), followed by “Psychotherapy focusing on changing thoughts and behaviors” (80.4%), and “Reading the Koran or Bible” (79.9%). “Psychotherapy” was considered the most helpful treatment by 38.6% of participants. “Anti-depressant medication” was the medication most commonly noted as helpful (79.6%), and was seen as the most helpful medication by 73.7% of participants. People cited as helpful were psychologists (80.7%), psychiatrists (78.2%), and community mental health workers (77.1%). Psychiatrist was cited as most helpful by 43.3% of participants.

**Table 3:** Perceived helpfulness of interventions for depression with suicidal thoughts vignette (*n* = 281)

Intervention	Helpful	Harmful	Neither	Most helpful^a^
Treatments and activities				
Getting information about the problem and available services	90.6	0.7	8.6	13.6
Psychotherapy focusing on changing thoughts and behaviours (cognitive behaviour therapy)	80.4	1.1	18.5	38.6
Reading the Koran or Bible	79.9	2.2	17.9	8.3
Getting out and about more/finding some new hobbies	70.4	2.2	27.4	0.8
Psychotherapy focusing on relationships with others	68.0	2.5	29.5	4.5
Psychotherapy focusing on causes that stem from the past	66.5	2.2	31.2	12.9
Relaxation (e.g. having a massage)	64.0	2.2	33.8	2.3
Just talking about the problem (e.g. to a family member or close friend)	62.9	5.8	31.3	5.3
Have a prayer session or reading with a religious leader	60.4	2.9	36.7	0.8
Improving diet and/or getting more exercise	55.4	1.8	42.8	1.5
Reading a self-help book	46.1	3.7	50.2	0.8
Admission to a psychiatric hospital	36.6	10.1	53.3	7.6
Hypnosis	25.0	6.7	68.3	0.0
Traditional therapies (e.g. herbs, honey, black cumin seed, olive oil, dates, cupping - Hijama)	18.3	8.6	73.1	1.5
Trying to deal with the problem on her own	15.2	34.9	49.8	1.5
Drinking alcohol to relax	7.6	69.8	22.7	0.0
Medicine Type				
Anti-depressant medication (e.g. Prozac)	79.6	3.3	17.1	73.7
Medication to help you relax (e.g. Xanax, Valium)	54.5	8.7	36.7	12.8
Vitamins and minerals (e.g. Vitamin C)	48.0	1.8	50.2	13.5
Person/Service				
Psychologist	80.7	0.7	18.5	22.4
Psychiatrist	78.2	2.5	19.3	43.3
Community mental health worker/team (e.g. social worker, mental health nurse)	77.1	1.8	21.1	6.0
Family member	70.2	0.4	29.5	7.5
Religious person or priest	68.8	0.7	30.4	8.2
Homeland social group/club	63.1	3.0	33.9	3.0
Close male friend	56.9	1.1	42.0	3.0
Family or local doctor	56.3	1.1	42.6	3.7
Community religious organization	50.0	2.2	47.8	1.5
Telephone counseling	37.9	5.5	56.6	0.0
Close female friend	34.8	2.9	62.3	1.5

^a^Percentage of sample rating the specific intervention item as ‘the most helpful’ for treating problem described in vignette

### Factors affecting responses to vignette questions

Participant’s scores on the K10 scale affected their recognition of depression. Specifically, participants with higher K10 scores (mean 23.7) selected the correct diagnosis (depression with suicidal thoughts) more frequently (*P* = 0.03). However, there was also a significant association between those with lower K10 scores and selection of the almost correct diagnosis of depression (*P* = 0.017).

### Psychosis: Saed

Participants were asked “What would you say is Saed’s main problem?” In total, 113 (54.3%) participants chose psychosis and 41 (19.7%) chose depression. In addition, 15 respondents (7.2%) thought Saed was suffering from anxiety. Collectively, this accounted for 81.3% of all responses.

Table 4 shows the percentage of respondents who considered interventions in each subcategory (treatment activities, medicines, or people) as “helpful,” “harmful,” or “neither” for the problem described. “Getting information” (87.4%), “Psychotherapy focusing on changing thoughts and behaviors” (78.6%), and “Psychotherapy focusing on relationships with others” (74.4%) were the treatment activities most commonly considered helpful. “Psychotherapy focusing on changing thoughts and behaviors” was the treatment activity considered most helpful by 41.4% of participants. “Anti-psychotic medicine” was the medication most commonly noted as helpful (76.0%), and was considered the most helpful medication by 49.5% of participants. Participants considered cited psychologists (83.9%), psychiatrists (83.7%) and community mental health workers (78.4%) as helpful people, and 46.6% thought a psychiatrist was most helpful.

**Table 4:** Perceived helpfulness of interventions for psychosis vignette (*n* = 208)

Intervention	Helpful	Harmful	Neither	Most Helpful^a^
Treatments and activities				
Getting information about the problem and available services	87.4	11.7	1.0	8.6
Psychotherapy focusing on changing thoughts and behaviours (cognitive behaviour therapy)	78.6	19.4	1.9	41.4
Psychotherapy focusing on relationships with others	74.4	22.7	2.9	2.9
Psychotherapy focusing on causes that stem from the past	73.0	25.5	1.5	7.1
Reading the Koran or Bible	72.5	25.5	2.0	5.7
Getting out and about more/finding some new hobbies	63.5	33.0	3.4	1.4
Relaxation (e.g. having a massage)	60.9	38.2	1.0	4.3
Admission to a psychiatric hospital	57.8	37.7	4.4	17.1
Have a prayer session or reading with a religious leader	56.8	42.2	1.0	2.9
Improving diet and/or getting more exercise	54.4	44.7	1.0	0.0
Just talking about the problem (e.g. to a family member or close friend)	52.7	39.8	7.5	2.9
Reading a self-help book	43.1	49.5	7.4	1.4
Hypnosis	37.1	56.2	6.7	2.9
Trying to deal with the problem on her own	26.3	39.0	34.6	1.4
Traditional therapies (e.g. herbs, honey, black cumin seed, olive oil, dates, cupping - Hijama)	23.6	71.4	4.9	0.0
Drinking alcohol to relax	6.3	22.2	71.5	0.0
Medicine Type				
Anti-psychotic medication (eg Seroquel)	76.0	22.5	1.5	49.5
Anti-depressant medication (e.g. Prozac)	65.4	32.2	2.4	24.2
Medication to help you relax (e.g. Xanax, Valium)	59.1	36.9	4.0	21.6
Vitamins and minerals (e.g. Vitamin C)	50.0	48.0	2.0	4.7
Person/Service				
Psychologist	83.9	0.0	16.1	27.4
Psychiatrist	83.7	1.0	15.3	46.6
Community mental health worker/team (e.g. social worker, mental health nurse)	78.4	1.0	20.6	5.5
Family member	64.0	2.5	33.5	4.1
Religious person or priest	61.2	3.4	35.4	2.7
Family or local doctor	55.9	1.0	43.1	2.7
Homeland social group/club	54.7	1.5	43.8	0.0
Close male friend	46.6	3.9	49.5	5.5
Community religious organization	45.0	2.5	52.5	2.7
Telephone counseling	36.6	5.9	57.4	1.4
Close female friend	24.5	5.0	70.5	1.4

^a^Percentage of sample rating the specific intervention item as ‘the most helpful’ for treating problem described in vignett

### Factors affecting responses to vignette questions

The only factor associated with the selection of the correct diagnosis of psychosis was the participant’s sex. Specifically, male participants more frequently selected anxiety as the diagnosis for this vignette, whereas females tended to select depression (*P* < 0.001).

## Discussion

The importance of optimal recognition and promotion of early and appropriate mental health treatment has consistently been demonstrated as associated with improved mental health outcomes [15]. The promotion of good mental health should encompass all healthcare professionals, rather than relying on mental healthcare providers alone. This study, which is the first of its kind in the UAE, sought to assess the MHL of healthcare professionals working with children and adolescents with chronic conditions. We found variation in the recognition of mental health disorders and beliefs regarding treatment that differed from the recommended practices taught in most health professional degrees based on a Western biomedical approach, and from those reported in research assessing levels of MHL in healthcare professional groups [21–23]. Although participants in this study represented a range of different cultures and may bring belief systems congruent with their cultural background to their clinical work, the UAE has largely adopted a Western biomedical approach to mental healthcare. In clinical settings, the DSM-V is used as the basis for diagnosis and treatment planning, and it is expected that participants would be familiar with this framework.

Specifically, our results demonstrated limited recognition of mental health disorders, ranging from 47% for PTSD to 54.3% for psychosis. Approximately half of the participants were unable to correctly identify the disorders described in the vignettes. This finding is concerning, particularly as it may result in potentially lethal consequences if the need for intervention is not identified (i.e., depression with suicidal thoughts). Research has demonstrated that identification or recognition of mental illness is associated with early and appropriate treatment seeking [15]. Therefore, our findings highlight the need for focused investment in mental health education and promotion programs for healthcare professionals in the UAE.

The low level of mental health knowledge shown in this study is congruent with other studies. A Nigerian study [24] that assessed healthcare professionals’ level of familiarity with mental health problems revealed that although a slight majority held positive attitudes toward persons with mental illness, many did not. The levels of education and knowledge about mental illness among participants in that study were significantly related to other factors such as prior exposure to reports in the media about mental health problems. Recommendations from that study included developing programs to improve health workers’ MHL and attitudes toward those with mental illness [24].

Other studies indicated that knowledge deficits also exist among specialized mental healthcare professionals, such as psychiatrists and nurses working in psychiatric healthcare settings [25, 26]. An assessment of MHL relating to eating disorders among 126 psychiatrists in the United Kingdom reported variable knowledge of eating disorders, with specific gaps in both diagnosis and management of eating disorders [25]. That study identified the need for focused education for psychiatrists regarding diagnosis and management of such disorders. Other recommendations to address identified knowledge deficits included implementing training programs and making information readily available [25].

Differences in MHL between healthcare professions may also impact the level of care provided. A Chinese study comparing psychiatrists’ and registered nurses’ levels of MHL in a Chinese general hospital identified psychiatrists as highly accurate in correctly diagnosing mental health problems, whereas registered nurses were less accurate [26]. Although the role of nurses in the UAE does not include the diagnosis of mental disorders, the ability to correctly identify and recognize signs and symptoms of major mental health problems is important in enabling nurses to appropriately refer patients to more specialized services. In the present study, the small number of participating medical doctors precluded comparisons of MHL between nurses and medical doctors in the UAE. However, differences in undergraduate education and training programs and opportunities for ongoing professional development for nurses and medical doctors may contribute to lower levels of MHL among nursing professionals.

The findings of this study have important implications for education and mental health promotion. The low levels of MHL in our sample suggest potential for healthcare service delivery to be compromised, especially in regard to identification and early treatment for children and young people with chronic physical health problems. This gap in knowledge requires attention from service planners and decision makers. A growing body of research demonstrates that focused training programs positively affect MHL [22, 27]. A US project evaluated a two-tiered training program to improve family physicians’ skills and confidence in diagnosing and treating patients with mental health problems. The program provided family physicians with training in adult mental health, and included mental health first aid training for medical office assistants. Those authors reported the training had a positive impact on participants’ clinical practice, and the behavior-change tools (provided as a training component) were effective [22]. Australian researchers reported on a longitudinal evaluation of changes in healthcare providers’ knowledge and beliefs regarding mental illness; following implementation of multiple national mental health promotion activities, there was improvement in MHL compared with the results of earlier studies (conducted between 1996 and 1997) [27].

The marked cultural diversity of the UAE population may require health professionals to be even more mindful of the potential influence of cultural factors on mental health issues. Expatriates comprise a large proportion of the UAE workforce. For many of the expatriate workforce, work or economic factors resulted in separation from families who remain in their homeland; many have families living in unstable regions of the world, or have themselves been displaced from their homelands for sociopolitical reasons. These factors contributed to the interest in exploring the participant’s levels of psychological distress as measured by their K10 scores and whether this influenced their MHL. Our findings indicated that 40% of participants scored moderate to severe levels of psychological distress. When exploring associations with MHL, it was noted that those with higher K10 scores were more likely to select the correct diagnosis when presented with the ‘depression with suicidal thoughts’ vignette. Further, an association between those with lower K10 scores and next most correct diagnosis of ‘depression’ was also noted for the same vignette. Previous studies of MHL in clinical populations such as traumatized refugees noted that levels of PTSD symptomology were not associated with improved recognition when participants were presented with a PTSD clinical vignette [17]. Further, given that no other associations were noted between psychological distress levels and the other vignettes in this study, this finding should be interpreted with caution and warrants the need for future replication.

In the UAE context, the patient population and the healthcare workforce are culturally heterogeneous. Only 3% of the UAE nursing workforce is Emirati nationals. Most non-Emirati clinicians completed their education in a range of different countries and educational systems. Consistent with the heterogeneity of the nursing workforce, it is expected that culturally-specific beliefs about mental health may impact on clinical practice. The potential impact of personal cultural and religious belief systems was explored in a qualitative study investigating the impact of South African Muslim general practitioners’ beliefs about mental health on their clinical practice [28]. Participants in that study held different religious beliefs from the majority of the South African population. That study analyzed the impact of clinicians’ belief systems on clinical practice in relation to mental illnesses, perceptions of the mental illness, the effect of religion and culture, and treatment of mental illness (including aspects of spiritual illness); the findings highlighted the need for greater awareness among these healthcare professionals regarding mental illness and an understanding of the differing religious and cultural taxonomies of illness held by the people to whom they provided care [29].

The present study demonstrated that MHL is low among the UAE healthcare workforce. Future research using a mixed methods approach should be undertaken to further elucidate the relationships between cultural beliefs and stigma and MHL levels in this population.

### Study limitations

This study has a number of limitations that should be noted. First, the length of the questionnaire (with three scenarios) in combination with a target audience of busy clinicians is likely to have reduced the response rate and number of fully completed surveys. The estimated 27% response rate may also limit the generalizability of the study results to the broader sample of UAE clinicians. Second, although healthcare professionals in the UAE are generally fluent in English, varying levels of English literacy might have influenced responses, particularly as limited funding and a small research team precluded administration of the questionnaires by bilingual researchers. Third, as this is the first study evaluating MHL among nursing and medical officers working in pediatric services in this region, no comparative data were available. Finally, the scenarios used in this survey study, although culturally adapted, were adult-based rather than child- or adolescent-focused. An important recommendation would be to conduct future research using vignettes that are specific to the target population, particularly since mental health issues in children can present differently than in adults.

### Conclusions and implications of the study

The richness and diversity of the UAE population presents opportunities and challenges for those responsible for the provision and delivery of healthcare. This study is the first of its kind in the UAE, and sought to determine the level of MHL among nurses and medical officers caring for children and young people presenting to pediatric services. The findings demonstrated that almost half of the participants were unable to accurately identify three mental illnesses (PTSD, depression with suicidal thoughts, and psychosis), and could not indicate the most appropriate care options. This highlights the need for curriculum enhancements for future health professionals and a targeted program of appropriate professional development focused on mental health promotion for those in clinical practice. Careful attention is required to ensure educational enhancements are culturally appropriate and support clinicians’ capacity to recognize the impact of their personal beliefs about mental illness on their practice, along with the diversity of religious and cultural beliefs of patients to whom they provide care.

The levels of psychological distress noted in this cohort also signals a need to ensure that appropriate supports are available for clinical staff employed in healthcare facilities; regular clinical supervision should be encouraged and peer support should be established to promote early and appropriate help seeking. Possible screening of clinicians in conjunction with ready access to programs designed to support mental wellbeing will develop and support the clinical workforce caring for children and adolescents. These findings have implications for future mental health promotion activities and education in the UAE and for other countries in the Gulf Cooperation Council region.
